# T-ECBM: a deep learning-based text-image multimodal model for tourist attraction recommendation

**DOI:** 10.1038/s41598-025-25630-z

**Published:** 2025-11-24

**Authors:** Jianfu Chen, Jiaxu Cong, Mingxiao Li, Yan Sun, Junying Zhang

**Affiliations:** 1 School of Intelligence Science and Engineering, Qinghai Minzu University, Xining, China; 2 School of Tourism, Qinghai Minzu University, Xining, China

**Keywords:** Deep learning, Multimodal fusion, Feature integration, Recommendation model, Tourist attractions, Environmental social sciences, Mathematics and computing

## Abstract

In recent years, tourism revenue and visitor numbers in Northwest China have increased steadily. However, many tourists still have limited knowledge of scenic destinations across the five northwestern provinces. When travelers intend to visit the region but have not yet decided on specific destinations, an intelligent recommendation system is urgently needed to assist their decision-making. Based on collaborative filtering, content matching, or knowledge graphs existing systems primarily face three major challenges: Due to reliance on historical data, the recommendation performance for new users and new attractions is weak; limited ability to capture tourists’ current intentions and personalized needs; insufficient utilization of multimodal information. To address these challenges, We propose a novel deep learning-based multimodal recommendation model, T-ECBM. A dataset comprising 23,488 user reviews and 4160 images of 52 attractions was collected. BERT was employed to extract semantic features from reviews, capturing subjective preferences and sentiment, while an improved EfficientNet-CA model extracted visual features from images to identify key scenic elements. The two feature sets were fused and fed into a multilayer perceptron, formulating the recommendation task as a multi-class classification problem. Experimental results demonstrate that text-only BERT achieved a Top-1 accuracy of 82.67%, while image-only EfficientNet-CA reached 83.68%. In contrast, the proposed T-ECBM achieved 96.71% Top-1 accuracy, 99.82% Top-5 accuracy, and an F1-score of 96.70%, proving its significant superiority over unimodal approaches. By integrating textual and visual modalities, T-ECBM effectively reduces information asymmetry, enriches decision-making support, and delivers intelligent, efficient, and personalized recommendations for tourists exploring northwestern China.

**Supplementary Information:**

The online version contains supplementary material available at 10.1038/s41598-025-25630-z.

## Introduction

With the rapid development of the Internet economy, online consumer reviews have become an important reference for enterprises to optimize products and improve service quality^1^. As key venues for tourism activities, scenic attractions provide facilities and services for visitors^2^. After visiting attractions, many tourists post reviews on online platforms. These reviews not only highlight existing problems—enabling managers to adjust and improve services—but also provide potential visitors with reference information, thereby helping them gain a better understanding of the attractions. Studies have shown that many tourists do not have predetermined destinations; instead, they browse tourism platforms, focusing on online reviews, scenic images, city locations, and price information to identify suitable attractions^3^.

In recent years, the tourism industry in Northwest China has experienced rapid growth, driven by its unique natural and cultural resources. Both tourism revenue and visitor numbers have steadily increased, positioning the region as an emerging destination of national significance. The area is home to numerous renowned attractions, including the Great Tang Everbright City, the Mausoleum of the First Qin Emperor, and the Tang Paradise in Shaanxi Province; Zhongshan Bridge, Crescent Spring at Singing Sand Dunes, and Zhangye Danxia in Gansu Province; Qinghai Lake, Chaka Salt Lake, and Ta’er Monastery in Qinghai Province; Shapotou and Zhenbeibao Western Film Studio in Ningxia Hui Autonomous Region; as well as the International Grand Bazaar, Kashgar Old City, and Sayram Lake in Xinjiang Uygur Autonomous Region. These sites attract large numbers of domestic tourists each year. Nevertheless, the region’s remote location and comparatively limited transportation infrastructure reduce its competitiveness in the short-haul tourism market. Its destinations remain less well-known than those in eastern China, and many travelers have limited awareness of or direct experience with Northwest scenic areas. When choosing destinations, tourists often struggle to articulate clear preferences or rely on landscape photographs taken by others, indicating a general lack of familiarity with specific destinations. This information asymmetry consequently hampers precise decision-making and highlights the need for more effective recommendation support.

To address the problems of information asymmetry and biased decision-making in tourism, this study proposes a deep learning-based multimodal recommendation model. It hold on tourism decision-making is inherently an emotion-driven process, requiring both visual stimulation and textual validation. The model address that a substantial semantic gap exists between textual and visual data, by jointly modeling multimodal features of image and textual semantics, capturing both emotional triggers and rational validation. Specifically, user-generated reviews reflect subjective evaluations and preferences, while scenic images convey intuitive visual impressions. Integrating the two not only compensates for the limitations of unimodal recommendation but also enhances accuracy and diversity. Given the multidimensional and complex nature of tourism information, such multimodal integration is particularly essential, as it reduces information asymmetry and provides more rich and reliable decision support for personalized attraction recommendation.

## Related work

### Applications of deep learning in tourism

Amid global digitalization and intelligence, deep learning of a core AI technology has been increasingly integrated into the tourism industry and has played a pivotal role in tourism research. For instance, a multimodal sarcasm detection model was constructed using deep learning, which extracted textual, emoji, and image features from online tourism reviews and integrated them through multiple fusion strategies^4^. A CNN-BiLSTM-CRF model was proposed to capture and represent local textual features, enabling the recognition of attraction entities^5^. An attention-based model was also developed to analyze online tourism texts, thereby enhancing tourist’s dining experiences in urban tourism contexts^6^. Furthermore, the image perception of forest parks was investigated by applying deep learning and user-generated content (UGC) image recognition, analyzing both cognitive and emotional dimensions to inform image construction and marketing strategies^7^. In addition, kernel density estimation, the Gini coefficient, geographic detectors, and deep learning methods were applied to examine the spatial distribution of high-level tourist attractions along the “Belt and Road” and to predict potential new regions suitable for tourism development^8^. Collectively, these studies demonstrate significant progress in integrating deep learning into tourism research, with applications primarily focused on sentiment analysis of reviews, tourism experience mining, scenic image recognition, and visitor behavior modeling. However, recommendation systems function as ex-ante decision-making tools that directly influence whether tourism activities occur, whereas image recognition and sentiment analysis are typically ex-post applied during or after the visit. Thus, recommendation systems not only integrate achievements from related fields but also face a much larger gap in deep learning applications. Their potential to drive digital transformation in tourism is greater than that of other domains.

### Deep learning-based tourism recommendation systems

Recommendation systems, as a subset of information filtering systems, aim to predict user preferences for items based on their interests, habits, personalized needs, and product attributes, thereby assisting users in decision-making and enhancing satisfaction^9^. Against the backdrop of digital transformation and the rapid growth of the tourism industry, tourism recommendation systems have become a prominent research focus. For example, personalized activity recommendations were generated using Twitter data by collecting users’ followed or bookmarked travel information^10^. Tourists’ travel personalities were modeled through semantic features extracted from attraction reviews^11^. Collaborative filtering was applied by integrating attraction tags such as region, time, theme, and type with user interests to improve recommendation accuracy^12^. A knowledge graph-based framework was developed to link recommendation processes with graph embeddings, enabling the inference of user interest propagation^13^. A gated recurrent unit-based trajectory mining model was proposed to generate personalized tour recommendations from historical visit paths^14^. Big data from online encyclopedias were also leveraged to analyze similarities between attractions for personalized recommendations^15^. In summary, existing research has relied heavily on deep data mining and machine learning algorithms to analyze tourists’ historical behaviors and preferences, enabling precise information delivery. However, most studies still focus on single-modality data, with limited exploration of multimodal fusion involving text and images. In tourism contexts, textual data encapsulates subjective preferences and emotional attitudes, while image data provides intuitive representations of scenic environments, landscapes, and atmospheres, both of which hold substantial value for recommendations.

Currently, mainstream approaches to attraction recommendation remain grounded in collaborative filtering, content matching, and knowledge graphs, yet they face three major limitations: (1) a heavy reliance on historical user data, resulting in poor performance for new users and attractions; (2) a limited capacity to capture tourists’ immediate intentions and personalized needs; and (3) a dependence on unimodal data sources. With its strengths in multimodal feature fusion and personalized modeling, deep learning offers a promising direction. Prior research has demonstrated the potential of multimodal integration to enhance recommendation accuracy. For example, a sentiment-aware model was developed that fused images and reviews, but it relied on early feature concatenation, which failed to capture deep cross-modal interactions^16^. Embeddings of visual and spatiotemporal embeddings were incorporated into attraction recommendation, though the method was constrained by its reliance on geotagged data^17^. Attention mechanisms were applied to decouple multimodal features, showing progress in product recommendation but lacking validation in real-world tourism contexts^18^. A heterogeneous dual-tower model for feature fusion improved performance on small- and medium-scale datasets, yet its scalability to large datasets remains untested^19^. A hypergraph neural network-based multimodal fusion model was also proposed to capture high-order associations between text and images, but its application has so far been limited to medicinal herb recognition rather than tourism recommendation^20^. Overall, existing studies have made some progress in image recognition and text modeling, but their application in tourism recommendation scenarios remains limited, especially for specific regions such as scenic spots in China’s five northwestern provinces. Nevertheless, these studies still provide valuable references for optimizing destination recommendation systems. Furthermore, existing efforts still face challenges including data collection constraints, complex feature extraction, and inconsistent fusion strategies.

## Data sources

### Textual data

In this study, the textual data used in this study were obtained from tourist reviews posted on the Ctrip travel platform (www.ctrip.com). Reviews were collected from 52 scenic attractions rated 5A or 4A in the five northwestern provinces of China, namely Shaanxi, Gansu, Ningxia Hui Autonomous Region, Qinghai, and Xinjiang Uygur Autonomous Region (hereafter referred to as the “Five Northwestern Provinces”). Using Python-based web scraping techniques^21^, between 240 and 500 reviews were gathered for each attraction, resulting in a total of 23,488 reviews. Data collection was completed on May 14, 2025.

### Image data

The image dataset was derived from user-uploaded review images on the Ctrip travel platform and supplementary images from Baidu Images (www.image.baidu.com). Images were collected for the same 52 scenic attractions in the Five Northwestern Provinces, with 80 images per attraction, yielding a total of 4160 images. Data collection was conducted on May 15, 2025. Each review text and image was labeled with a unique scenic attraction category. For reproducibility and to facilitate international readers’ understanding, an appendix provides a bilingual (Chinese–English) reference table listing the names of all 52 scenic attractions.

## Model construction

### Text-based recommendation model

#### BERT-based model

**Fig. 1:**
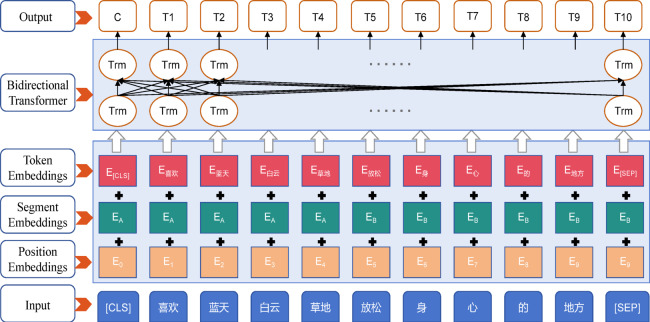
Input representation and network architecture of the BERT model.

BERT (Bidirectional Encoder Representations from Transformers)^22^ is a pre-trained language model built on a bidirectional Transformer encoder (Fig. 1). For scenic attraction review data, we employed a Chinese BERT model, fine-tuned on 23,488 reviews from the 52 attractions, to extract semantic textual features.

#### Chinese word segmentation

Unlike English text, Chinese text does not use spaces or explicit markers to delimit words, requiring segmentation into meaningful word sequences^23,24^. The WordPiece tokenizer of BERT divides text into characters and common multi-character phrases. For example, the sentence “喜欢蓝天白云草地, 放松身心的地方” (“like blue sky, white clouds, and grassland, a place to relax”) would be tokenized as ([CLS], “喜欢”, “蓝天”, “白云”, “草地”, “放松”, “身”, “心”, “的”, “地方”, [SEP]). Here, [CLS] and [SEP] are special control tokens placed at the beginning and end of the sequence, which is then input into BERT (Fig. 1).

#### Word embedding representation

BERT converts segmented tokens into vector representations through three embeddings: token embeddings (semantic representation), segment embeddings (sentence-level distinction), and position embeddings (absolute position in the sequence). The sum of these three embeddings forms the final input representation for the model.

#### Bidirectional transformer encoder

The embedded sequence is processed by a bidirectional Transformer encoder, which leverages self-attention to capture contextual dependencies among tokens. For the example input sequence, BERT outputs a set of vectors {C, T1, T2, … T10}, where C represents the holistic semantic feature of the review, and T1–T10 represent contextual features of individual tokens.

### Image-based recommendation model

#### EfficientNet-based model

**Fig. 2:**
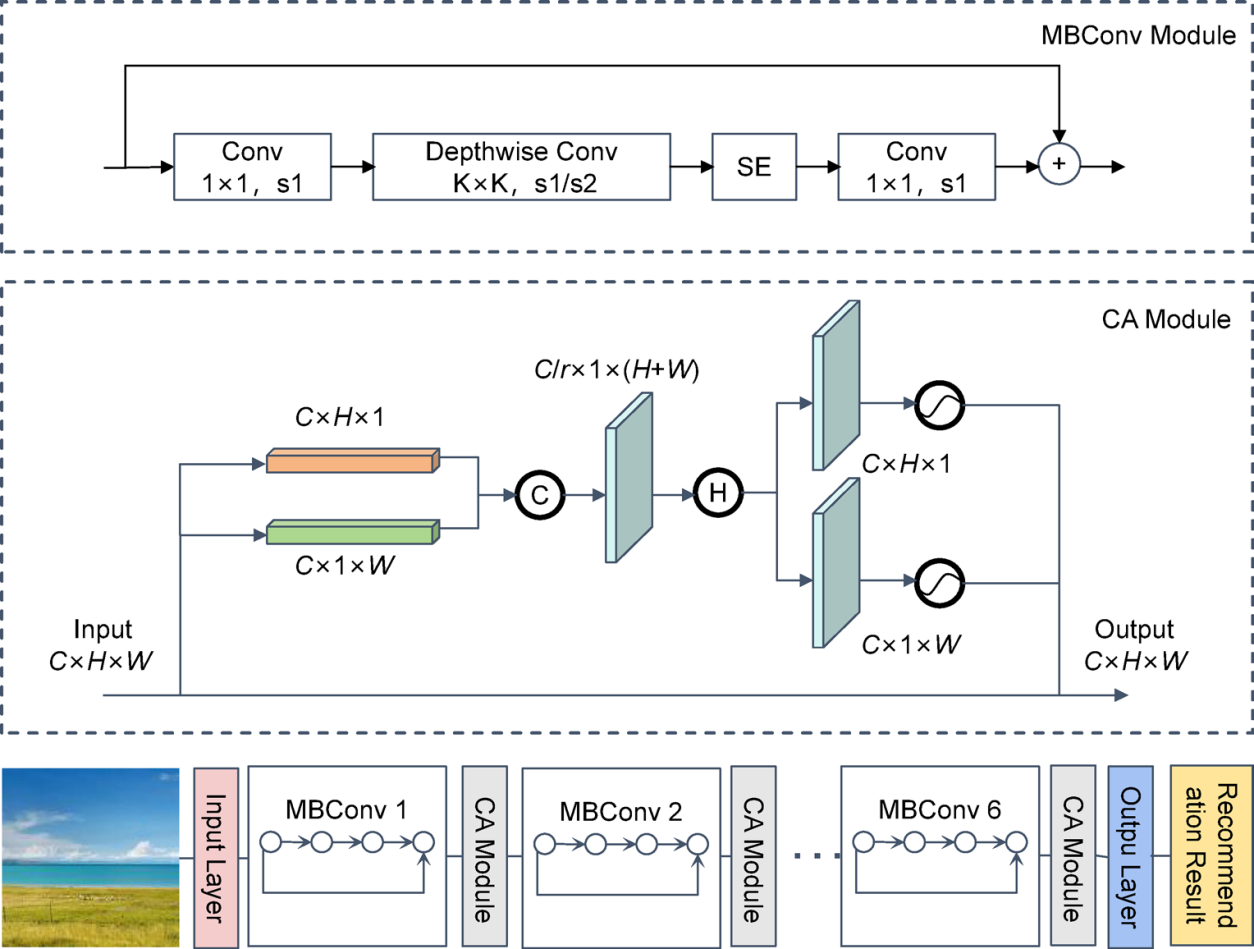
Network architecture of EfficientNet_CA and its main modules.

To extract visual features of scenic images, we adopted the EfficientNet-B0 architecture^25^. EfficientNet utilizes a compound scaling method that simultaneously optimizes the network’s depth, width, and resolution. Its design integrates depthwise separable convolutions^26^, squeeze-and-excitation (SE) modules^27^, and residual connections^28^. The architecture consists of an initial convolution layer, seven MBConv blocks, and a classification head (Fig. 2).

Among the various convolutional neural network architectures, EfficientNet-B0 was selected in this study for several reasons. First, as the baseline model of the EfficientNet family, B0 has relatively fewer parameters and lower computational cost, making it suitable for large-scale scenic image feature extraction under limited computational resources. Second, compared with commonly used architectures such as ResNet, DenseNet, and Inception, previous studies have demonstrated that EfficientNet-B0 achieves comparable or even superior accuracy on multiple vision tasks (e.g., image classification and scene recognition) with significantly lower computational overhead^29,30^. Furthermore, in the context of tourism image recommendation, the model must balance both accuracy and efficiency to ensure scalability in real-world applications. Therefore, EfficientNet-B0 strikes an appropriate trade-off between performance and computational cost, making it the optimal choice for this study. It should be noted, however, that with more abundant computational resources, larger variants of EfficientNet could potentially achieve even higher accuracy.

#### Coordinate attention mechanism

Recent advances have underscored the effectiveness of attention mechanisms in visual tasks^31^. To this end, Hou introduced the Coordinate Attention (CA) module^32^, which extends beyond SE and CBAM by encoding direction-aware spatial information. As illustrated in Fig. 2, CA applies pooling operations along the height (H) and width (W) dimensions to generate direction-aware feature maps, thereby enabling the model to capture long-range dependencies while retaining precise positional information.

#### Improved EfficientNet_CA model

To further enhance the performance of EfficientNet in scenic image feature extraction, this study incorporates a Coordinate Attention (CA) module into the original architecture and proposes the EfficientNet_CA network as the image feature extraction component. As illustrated in Fig. 2, a CA module is embedded after each MBConv block of EfficientNet. The improved EfficientNet_CA model not only preserves the efficient feature extraction capability of the original architecture but also strengthens spatial information encoding and inter-channel dependency modeling through the CA mechanism.

### Image–text recommendation model

In this study, the scenic spot recommendation task is formulated as a multi-class classification problem. Specifically, the output layer of the model is set to 52 categories, corresponding to the 52 scenic spots across the five northwestern provinces of China. During training, the cross-entropy loss function (CrossEntropyLoss) is adopted to optimize the model by minimizing the discrepancy between the predicted probability distribution and the ground-truth labels. In the inference phase, the model generates a probability distribution over all scenic spots, where the Top-1 recommendation corresponds to the category with the highest probability, and the Top-5 recommendations correspond to the top five categories with the highest probabilities. The proposed recommendation mechanism relies solely on content-driven classification of textual and visual inputs, without incorporating users’ historical behavior or personalized profiles. Thus, the essence of this study’s recommendation is to map the user’s input to the most likely scenic spot category, and then generate a ranked list of candidate spots. This content-based approach simplifies the system by avoiding the complexities of user modeling and personal data collection, leading to a more efficient recommendation process. It provides users with multiple potential options even when they have not yet determined a specific destination, thereby narrowing their choices and facilitating decision-making.

#### Feature fusion

For textual feature extraction, a pre-trained BERT model is employed. Since each scenic spot has approximately 80 images but 200–500 comments, to improve alignment between text and image data, every two comments are concatenated and used as one input to the BERT model. This yields textual feature vectors with a shape of 4160 × 768, where 4160 corresponds to the number of input samples and 768 is determined by the BERT architecture.

For visual feature extraction, the improved EfficientNet_CA model is used. Its classification layer is removed, and the feature output before the fully connected layer is retained, resulting in image feature vectors of shape 4160 × 1280, where 4160 corresponds to the number of input samples and 1280 to the EfficientNet-B0 architecture.

**Fig. 3:**
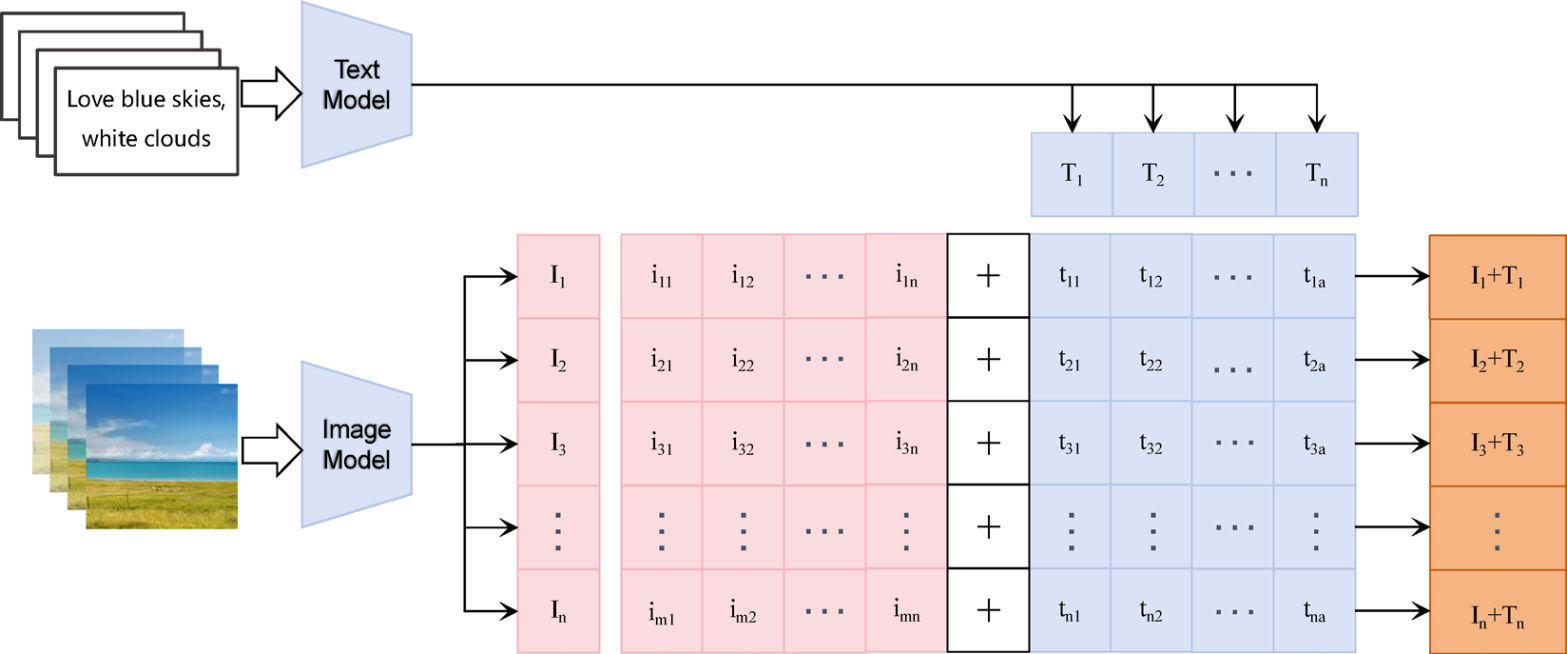
Process of text-image feature fusion.

Feature fusion is achieved using a concatenation (CAT) method^33^, as shown in Fig. 3. Specifically, the image feature vector I (4160 × 1280) and the text feature vector T (4160 × 768) are concatenated along the feature dimension, producing a multimodal representation vector of shape 4160 × 2048. This fused vector is then used as the input to the subsequent multilayer perceptron (MLP).

#### Multilayer perceptron

The multilayer perceptron (MLP)^34^ is one of the most fundamental and widely used neural network architectures, commonly applied in classification and regression tasks. It consists of an input layer, one or more hidden layers, and an output layer, where neurons between adjacent layers are fully connected. Each neuron performs a linear transformation followed by a nonlinear activation function, enabling the network to capture complex patterns.

**Fig. 4:**
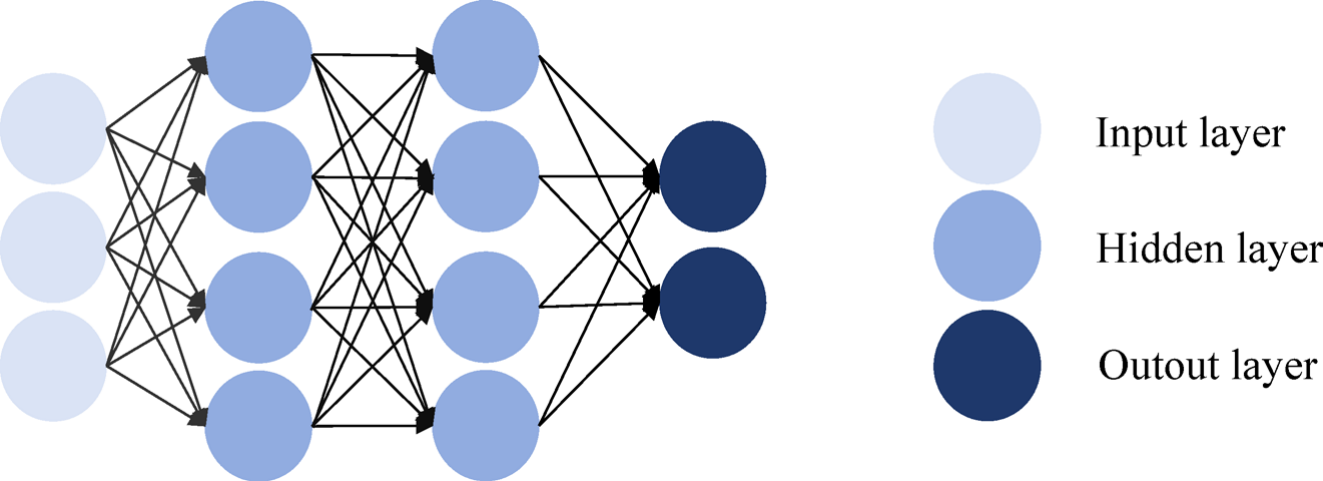
Schematic diagram of the MLP structure.

As illustrated in Fig. 4, the MLP designed in this study takes the fused 2048-dimensional multimodal feature vector as input, which is progressively mapped into lower-dimensional semantic spaces through two hidden layers, and finally projected into the class space by the output layer^35^. Let $h^{\left( 0 \right)}$ denote the input vector, the output of the $l$-th hidden layer is computed as:

1 $$h^{\left( l \right)} = ReLU\left( {W^{\left( l \right)} h^{{\left( {l - 1} \right)}} + b^{\left( l \right)} } \right)$$

Among them,$h^{{\left( {l - 1} \right)}}$ is the output of the previous layer,$W^{\left( l \right)}$ and $b^{\left( l \right)}$ are the layer’s weight matrix and bias vector, respectively, $ReLU$ is the activation function. Finally, the output layer maps the features to the category space to realize the recommendation of tourist scenic spots.

#### T-ECBM multimodal recommendation model

**Fig. 5:**
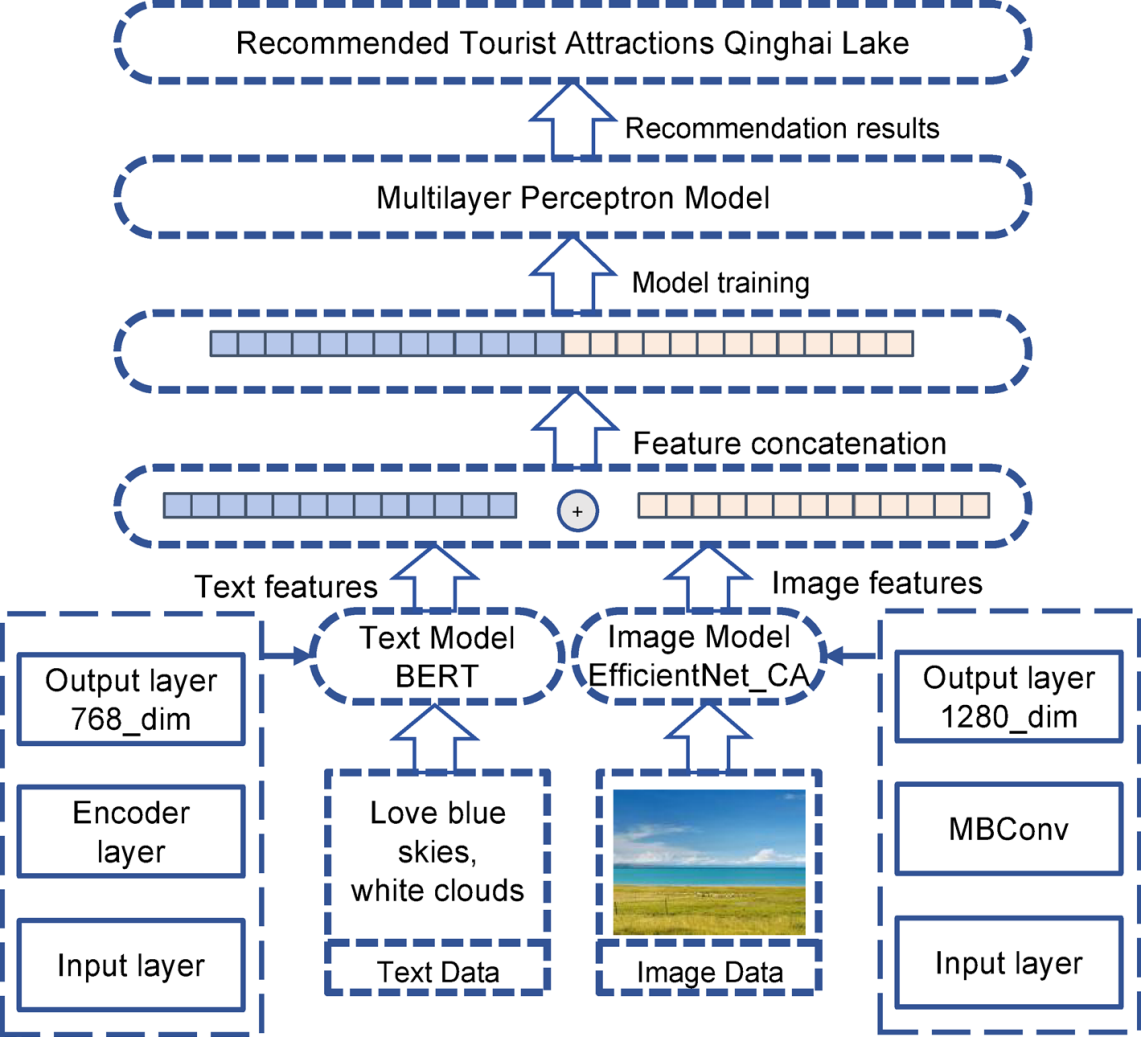
The proposed deep learning-based text-image multimodal tourist attraction recommendation model (T-ECBM).

In summary, this study proposes a deep learning-based multimodal scenic spot recommendation model, T-ECBM. As shown in Fig. 5, the model consists of four core modules: the BERT-based text feature extractor, the EfficientNet_CA image feature extractor, the feature concatenation module, and the MLP classifier. The overall framework treats the recommendation task as a multi-class classification problem, aiming to predict the most likely scenic spot category from the given text and image inputs. The recommendation results (e.g., “Qinghai Lake”) directly correspond to the predicted scenic spot category, and the final ranked list is generated by probability sorting. This design realizes a purely content-driven recommendation mechanism for scenic spots.

## Experimental results and analysis

### Experimental setup

The experiments were conducted on the AutoDL platform, utilizing an RTX 4090D GPU with 24 GB of VRAM and a 16-core Intel Xeon Platinum 8481C CPU, with 80 GB of system memory. The software environment was built on Ubuntu 22.04, using Python 3.12 in combination with the PyTorch 2.3.0 deep learning framework and CUDA 12.1 for model training and evaluation.

To ensure reliability and comparability, all models were trained under identical configurations. The experiments used a unified tourism scenic spot multimodal dataset, split into training and testing sets with an 8:2 ratio, and employed fivefold cross-validation. Results are reported as mean ± standard deviation. Statistical significance of differences was assessed using paired t-tests (*p* < 0.05). The specific experimental settings were as follows: batch size of 32, 50 training epochs, a fixed random seed of 42, CrossEntropyLoss as the loss function for multi-class classification, and the Adam optimizer with an initial learning rate of 0.001. A StepLR learning rate decay strategy was applied, reducing the learning rate by a factor of 0.7 every 10 epochs to improve stability and convergence. Evaluation metrics included Top-1 accuracy, Top-5 accuracy, and F1 score, with model parameter counts also considered to assess model scale and complexity comprehensively.

### Comparative experiments of text recommendation models

To evaluate the performance of different text modeling approaches in the tourism recommendation task, four representative natural language processing models were compared: Recurrent Neural Network (RNN), Long Short-Term Memory network (LSTM), Gated Recurrent Unit (GRU)^36,37^, and the pre-trained language model BERT. All models were trained on the same dataset with identical hyperparameters using fivefold cross-validation. Evaluation metrics included Top-1 accuracy, Top-5 accuracy, and F1 score, while the number of trainable parameters was also compared to assess performance versus resource consumption. The results are shown in Table 1.

**Table 1:** Experimental results of text models.

Model	Number of parameters	Top-1 accuracy (%)	Top-5 accuracy (%)	F1-score (%)
RNN	663,348	80.52 ± 0.76	92.14 ± 0.35	80.35 ± 0.75
LSTM	2,633,268	80.57 ± 0.67	92.34 ± 0.40	80.40 ± 0.66
GRU	1,976,628	80.54 ± 0.45	92.22 ± 0.39	80.38 ± 0.45
BERT	109,522,228	82.67 ± 0.42	92.52 ± 0.41	82.60 ± 0.42

As shown in Table 1, the BERT model achieved the highest performance across all metrics, with a Top-1 accuracy of 82.67 ± 0.42%, Top-5 accuracy of 92.52 ± 0.41%, and F1 score of 82.60 ± 0.42%. Paired t-tests revealed that the differences between BERT and the other three sequential models in Top-1 and F1 metrics were statistically significant (*p* < 0.05), confirming BERT’s superior capability in semantic understanding and context modeling, particularly for subjective and descriptively rich tourism reviews.

Regarding resource efficiency, the RNN model had the smallest parameter count (0.66 M) while still achieving a Top-1 accuracy of 80.52 ± 0.76% and an F1 score of 80.35 ± 0.75%. Its performance was not significantly different from LSTM and GRU (*p* > 0.05), demonstrating a favorable balance between accuracy and efficiency, which makes it suitable for deployment in computationally constrained environments. Considering both accuracy and computational cost, BERT was selected as the high-precision representative for subsequent multimodal experiments, while RNN was chosen as a lightweight benchmark to evaluate text-image fusion under different resource constraints.

**Fig. 6:**
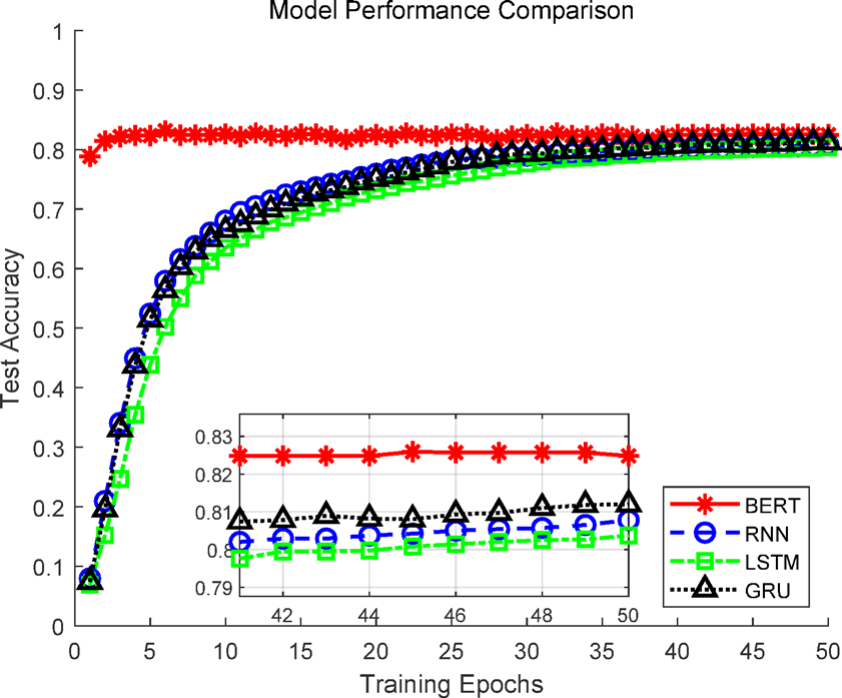
Accuracy curves of the text models.

**Fig. 7:**
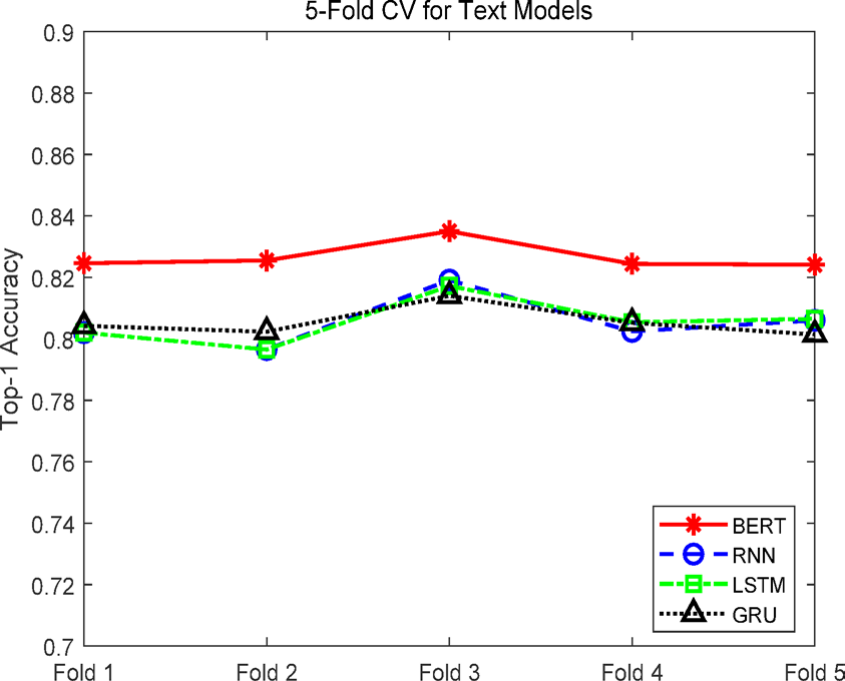
Fivefold cross-validation curves of the text models.

As shown in Fig. 6, BERT demonstrated clear superiority during training, with test Top-1 accuracy gradually approaching saturation while maintaining high levels. This indicates strong convergence capability and consistent performance improvement across multiple epochs. In contrast, RNN, LSTM, and GRU showed lower test accuracy and slower convergence, with incremental improvements over epochs that still did not reach BERT’s performance. Figure 7 further illustrates that BERT consistently outperformed the other models across all five folds in cross-validation, confirming its stability and high performance. RNN, LSTM, and GRU exhibited greater variability across folds, with GRU being the most unstable.

### Comparative experiments of image-based recommendation models

To evaluate the performance of different image models in tourism recommendation, four mainstream convolutional neural network architectures were compared: GoogLeNet, ResNet-152, DenseNet-121^38,39^, and EfficientNet, all enhanced with coordinate attention mechanisms. All models were trained with the same dataset and hyperparameter settings using fivefold cross-validation. Evaluation metrics included Top-1 accuracy, Top-5 accuracy, and F1 score, along with trainable parameter counts to assess performance versus resource usage. Results are summarized in Table 2.

**Table 2:** Experimental results of image models.

Model	Number of parameters	Top-1 accuracy (%)	Top-5 accuracy (%)	F1-score (%)
GoogLeNet_CA	6,058,580	83.13 ± 1.76	95.43 ± 0.94	82.75 ± 1.75
ResNet-152_CA	58,864,278	82.29 ± 0.85	94.11 ± 0.93	81.90 ± 0.85
DenseNet-121_CA	7,240,380	83.16 ± 0.87	96.20 ± 0.36	82.78 ± 0.87
EfficientNet_CA	4,230,440	83.68 ± 1.13	97.07 ± 0.71	83.28 ± 1.12

The EfficientNet_CA model achieved the highest performance across all metrics, with a Top-1 accuracy of 83.68 ± 1.13%, Top-5 accuracy of 97.07 ± 0.71%, and F1 score of 83.28 ± 1.12%. Paired t-tests confirmed that improvements in Top-1 and F1 scores over the other models were statistically significant (*p* < 0.05), validating EfficientNet_CA’s superiority in tourism image recognition tasks. Notably, EfficientNet_CA’s parameter count was only 4.23 M, the lowest among the four models, demonstrating its exceptional efficiency and resource utilization while maintaining high accuracy.

Among the other models, GoogLeNet_CA and DenseNet-121_CA showed slightly lower Top-1 accuracy and F1 scores compared to EfficientNet_CA but remained competitive in Top-5 accuracy, with moderate parameter counts offering deployment advantages. ResNet-152_CA, despite having the largest parameter count (58.86 M), achieved only 82.29 ± 0.85% Top-1 accuracy and 81.90 ± 0.85 F1 score, indicating limited potential exploitation, likely due to overfitting or feature redundancy.

**Fig. 8:**
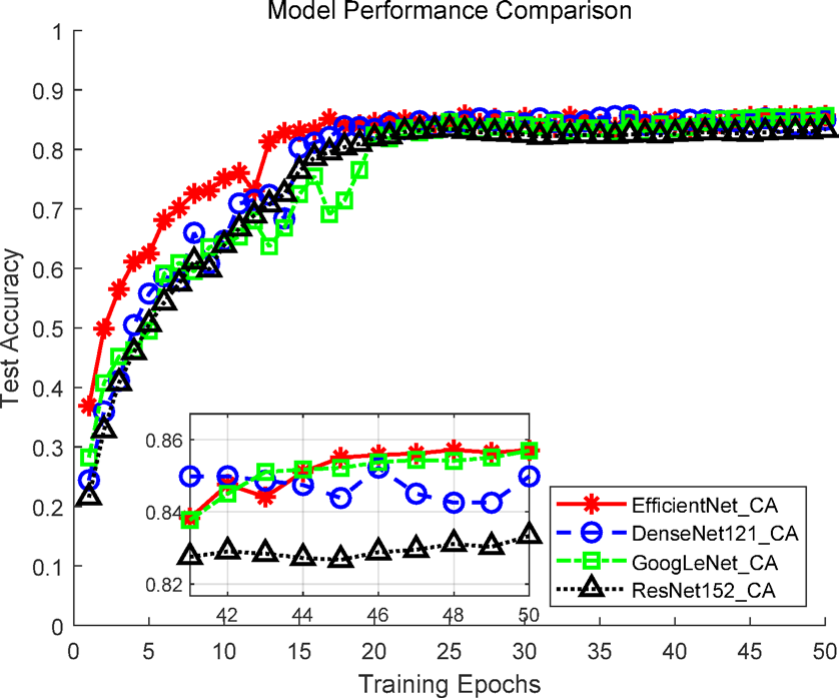
Accuracy curves of the image models.

Considering both accuracy and computational cost, EfficientNet_CA, GoogLeNet_CA, and DenseNet-121_CA were selected for subsequent multimodal experiments to evaluate performance across different architectures and scales. As shown in Fig. 8, EfficientNet_CA exhibited rapid early training progress, with test Top-1 accuracy quickly approaching saturation, reflecting strong convergence capability. DenseNet-121_CA and GoogLeNet_CA demonstrated slightly slower accuracy growth but maintained stable trends, showing overall robustness. ResNet-152_CA showed slow accuracy improvement and failed to match the performance of other models, likely due to its high parameter count limiting task-specific effectiveness.

**Fig. 9:**
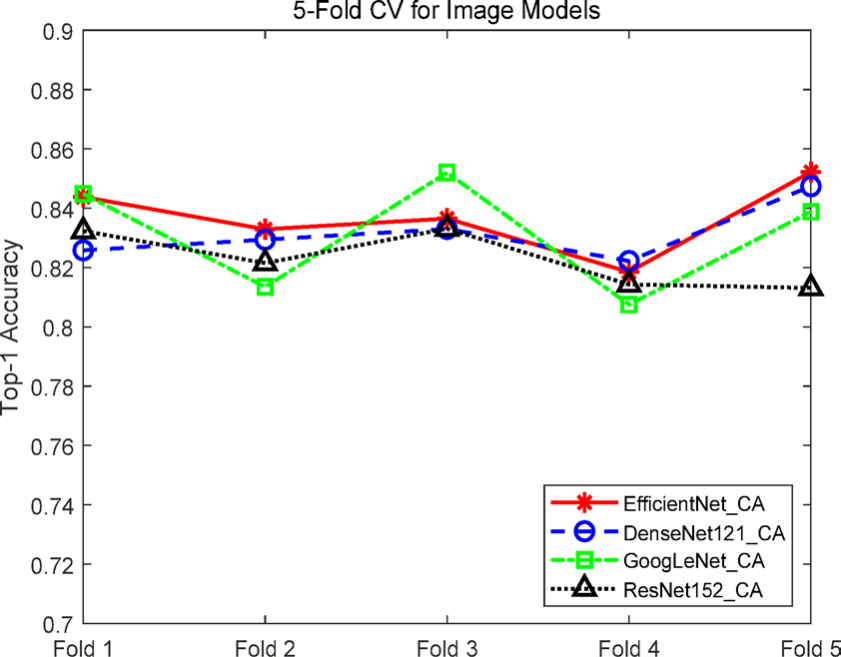
Fivefold cross-validation curves of the image models.

Figure 9 illustrates EfficientNet_CA’s stable performance across the five folds, with Top-1 accuracy in folds 1, 2, and 5 consistently exceeding the other models, confirming its strong generalization across different data splits. DenseNet-121_CA and GoogLeNet_CA displayed similar performance with moderate fluctuations, maintaining high overall levels. ResNet-152_CA exhibited the greatest variability, particularly in threefold and fivefold, with the lowest overall performance, highlighting the overfitting risk associated with its large parameter count.

### Comparative experiments of text–image fusion models

To comprehensively evaluate the performance of the proposed text-image multimodal recommendation framework, multiple combinations of fusion models were constructed for comparative experiments. The text feature extraction module employed either RNN or BERT, while the image feature extraction module used GoogLeNet_CA, DenseNet-121_CA, or EfficientNet_CA. The extracted text and image features were concatenated and input into a multilayer perceptron (MLP) for final classification. All experiments were conducted on the same dataset with identical hyperparameter settings using fivefold cross-validation. Evaluation metrics included Top-1 accuracy, Top-5 accuracy, and F1 score. Experimental results are summarized in Table 3.

**Table 3:** Experimental results of text-image fusion models.

Model	Top-1 accuracy (%)	Top-5 accuracy (%)	F1-score (%)
GoogLeNet + CA + RNN + MLP	88.52 ± 1.15	98.25 ± 0.48	88.15 ± 1.15
GoogLeNet + CA + BERT + MLP	94.01 ± 1.24	99.12 ± 0.20	93.65 ± 1.24
DenseNet-121 + CA + RNN + MLP	91.51 ± 1.04	98.85 ± 0.47	91.10 ± 1.04
DenseNet-121 + CA + BERT + MLP	96.20 ± 0.71	99.69 ± 0.30	95.75 ± 0.73
EfficientNet + CA + RNN + MLP	94.69 ± 0.41	98.87 ± 0.25	94.25 ± 0.41
EfficientNet + CA + BERT + MLP (T-ECBM, ours)	96.71 ± 0.43	99.82 ± 0.26	96.70 ± 0.44

The results show that BERT as the text feature extractor consistently outperformed RNN across all fusion combinations. Paired t-tests indicate that compared to RNN-based models, BERT-based combinations achieved statistically significant improvements in Top-1, Top-5, and F1 scores (*p* < 0.05), with an average Top-1 improvement of 4.07%. In the image branch, EfficientNet_CA achieved statistically significant improvements over GoogLeNet_CA and DenseNet-121_CA in all metrics (*p* < 0.05), with an average Top-1 increase of 4.4% compared to GoogLeNet_CA and 1.8% compared to DenseNet-121_CA. These results highlight EfficientNet_CA’s superior feature extraction and discriminative capabilities while maintaining a lightweight design.

Considering both modalities, the combination EfficientNet + CA + BERT + MLP (the proposed T-ECBM model) achieved the best performance across all metrics, with a Top-1 accuracy of 96.71 ± 0.43%, Top-5 accuracy of 99.82 ± 0.26%, and F1 score of 96.70 ± 0.44. Paired t-tests confirmed that these improvements were statistically significant compared to all other combinations. In resource-constrained deployment scenarios, EfficientNet + CA + RNN + MLP still achieved a Top-1 accuracy of 94.69 ± 0.41% and an F1 score of 94.25 ± 0.41 with fewer parameters, offeringquery practical advantages.

**Fig. 10:**
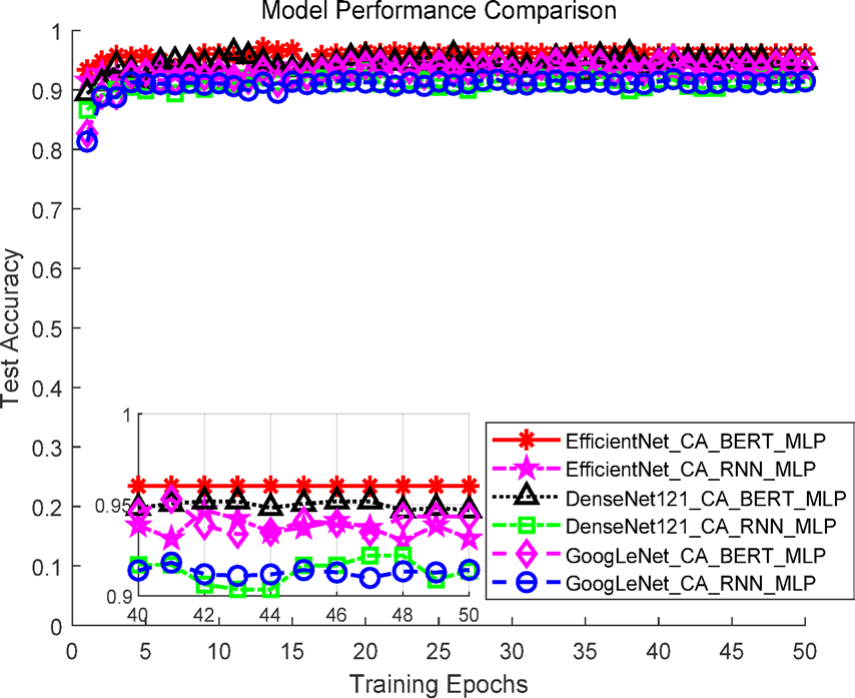
Accuracy curves of the text-image fusion models.

**Fig. 11:**
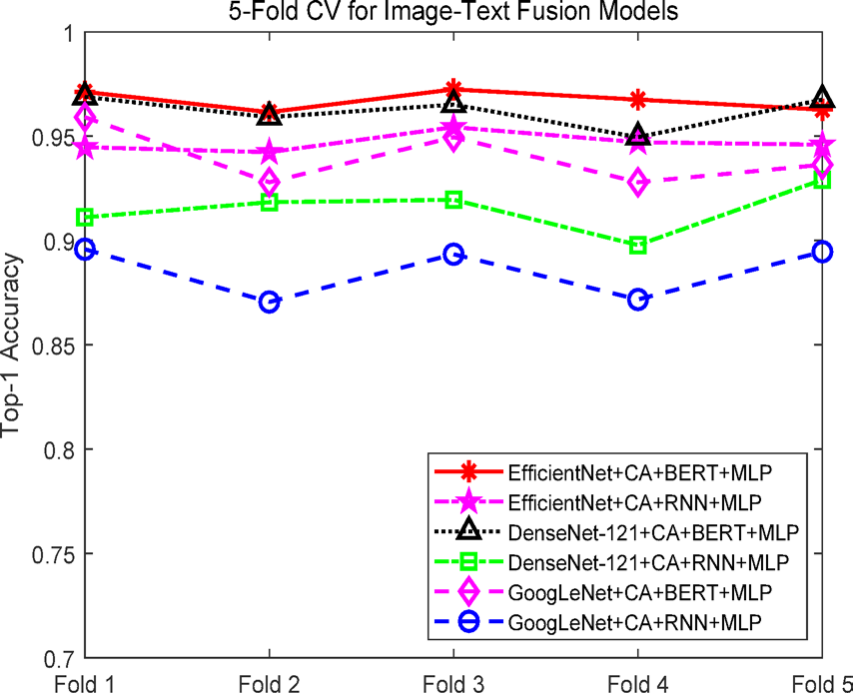
Fivefold cross-validation curves of the text-image fusion models.

As shown in Fig. 10, the test Top-1 accuracy curves during training indicate that the T-ECBM model consistently outperformed other combinations from the early training stages and maintained its lead throughout. GoogLeNet + CA + BERT + MLP and DenseNet-121 + CA + BERT + MLP also showed strong performance, though their accuracy curves exhibited slightly more fluctuation compared to T-ECBM. Figure 11 further illustrates that T-ECBM maintained the highest performance with minimal variance across all five folds of cross-validation, demonstrating high stability and strong generalization. In contrast, other fusion models exhibited varying degrees of accuracy fluctuation across folds and consistently performed below T-ECBM.

### Ablation experiments

To verify the effectiveness of each key module in the proposed framework, a series of ablation experiments were conducted, sequentially evaluating the impact of the text modality, image modality (before and after improvement), and text-image multimodal fusion on overall performance. All experiments were performed on the same dataset with identical hyperparameter settings using fivefold cross-validation, and paired t-tests (*p* < 0.05) were applied for statistical significance analysis. Evaluation metrics included Top-1 accuracy, Top-5 accuracy, and F1 score. Results are presented in Table 4.

**Table 4:** Results of ablation studies.

Model	Top-1 accuracy (%)	Top-5 accuracy (%)	F1-score (%)
BERT	82.67 ± 0.42	92.52 ± 0.41	82.60 ± 0.42
EfficientNet	81.61 ± 1.25	96.51 ± 0.78	81.20 ± 1.17
EfficientNet_CA	83.68 ± 1.13	97.07 ± 0.71	83.28 ± 1.12
T-ECBM	96.71 ± 0.43	99.82 ± 0.26	96.70 ± 0.44

The results show that using only the text modality (BERT), the model achieved a Top-1 accuracy of 82.67 ± 0.42% and an F1 score of 82.60 ± 0.42%, indicating that textual review information can capture semantic features of scenic spots effectively but still has limitations. Using the unmodified image modality (EfficientNet) alone yielded a Top-1 accuracy of 81.61 ± 1.25% and an F1 score of 81.20 ± 1.17%, demonstrating comparable performance and highlighting the importance of visual information in capturing scenic features.

When the coordinate attention mechanism (CA) was introduced in the image branch, EfficientNet_CA achieved a Top-1 accuracy of 83.68 ± 1.13% and an F1 score of 83.28 ± 1.12%, showing statistically significant improvements over the unmodified EfficientNet. This indicates that the CA module effectively enhances the representation of key regions in images.

Building on this, the proposed T-ECBM model (EfficientNet + CA + BERT + MLP) achieved a substantial performance leap through text-image multimodal fusion, with a Top-1 accuracy of 96.71 ± 0.43%, Top-5 accuracy of 99.82 ± 0.26%, and F1 score of 96.70 ± 0.44. These gains were statistically significant compared to single-modality models, confirming the advantages of the multimodal fusion strategy. The results demonstrate that simultaneously leveraging text and image features can significantly enhance the accuracy and robustness of tourism recommendation systems.

## Analysis of tourism recommendation results

### Recommendation results using BERT

The BERT model was employed to recommend tourist attractions (see Table 5). For Participant 1, the input text was “Visiting red tourism sites to experience patriotic feelings”. Among the top five recommended destinations, four were related to red culture, and all five were located in Yan’an, Shaanxi Province—a key revolutionary base and a well-known red tourism destination in China. These results demonstrate high credibility and accuracy.

**Table 5:** Recommendation results of the BERT model.

Text input	Tourist attraction recommendations (Top 1–Top 5)				
Visiting red tourism sites to experience patriotic feelings	Baota Mountain of Yan’an	Zaoyuan Revolutionary Site	Yangjialing Revolutionary Site	Yan’an Revolutionary Memorial Hall	Mausoleum of Yellow Emperor
Planning to travel with my child and parents	Qujiang Ocean Park	Lanzhou Polar Ocean World	Qinling Wildlife Park	Xining Wildlife Park	Jiayuguan Fortress
Prefer places with blue skies, white clouds, and green grass to relax	Kalajun Grassland Scenic Area	Nalati Scenic Area	Urumqi Tianshan Grand Canyon	Bayanbulak Scenic Area	Jiangbulake Scenic Area
Low budget, high cost-performance, not pursuing luxury	Yan’an Revolutionary Memorial Hall	Mount Huashan	Xining Wildlife Park	Shaanxi History Museum	Jiayuguan Fortress
Hope for enthusiastic, attentive, and detailed guided explanations	Yan’an Revolutionary Memorial Hall	Qinghai Provincial Museum	Zaoyuan Revolutionary Site	Gansu Provincial Museum	Famen Temple Scenic Area
Want to experience performances or exhibitions reflecting local cultural characteristics	Xinjiang Khanma Site	Grape Valley	Guane Valley Scenic Area	Qinghai Provincial Museum	Shuidonggou Tourist Area

Participant 2 input “Planning to travel with my child and parents”. The top four recommended sites were all zoos, which are representative leisure attractions suitable for family outings and appealing to children, indicating strong recommendation relevance.

Participant 3 entered “Prefer places with blue skies, white clouds, and green grass to relax”. The recommended destinations were concentrated in Xinjiang Uygur Autonomous Region, China’s largest province with abundant natural resources and diverse landscapes. During summer, it is a popular destination for relaxation and outdoor leisure, showing that the model’s recommendations align well with the visitor’s intent.

Participant 4 input “Low budget, high cost-performance, not pursuing luxury”. Among the top five recommendations, two were museums with public accessibility and free entry, while the other three focused on natural landscapes with extended sightseeing time, satisfying the visitor’s requirement for high cost-performance.

Participant 5 entered “Hope for enthusiastic, attentive, and detailed guided explanations”. Three of the recommended attractions were museums equipped with professional guides, matching the visitor’s expectations.

Participant 6 input “Want to experience performances or exhibitions reflecting local cultural characteristics”. The recommended destinations included two sites in Xinjiang, one in Ningxia Hui Autonomous Region, and one in Qinghai Province—all regions rich in ethnic minority culture, offering insights into the unique customs and activities of Northwest China, aligning closely with the visitor’s preferences.

Overall, across six participants and different dimensions such as tourism theme, audience type, attraction type, cost, services, and cultural activities, the T-ECBM model consistently provided recommendations that matched visitors’ideal attractions, demonstrating its ability to accurately identify and satisfy diverse user needs.

### Recommendation results using EfficientNet_CA

The EfficientNet_CA model was used for image-based tourism recommendation (see Table 6). Participant 7 uploaded a photo taken in July 2024 on the eastern shore of Qinghai Lake. The model ranked Qinghai Lake as the top recommendation, consistent with the visitor’s expectation.

**Table 6:** Recommendation results of the EfficientNet_CA model.

Image input	Tourist attraction recommendations (Top1–Top5)				
	Qinghai Lake	Sayram Lake	Chaka Salt Lake	Bayanbulak Scenic Area	Sand Lake Ecotourism Area
	Gansu Provincial Museum	Qinghai Provincial Museum	Shaanxi History Museum	Forest of Stone Steles Museum	Qin Shi Huang Mausoleum Museum
	Taer Monastery	Kongtong Mountain	Famen Temple Scenic Area	The Xi’an Circumvallation	Kashgar Old Town
	Qingtongxia Yellow River Grand Canyon	Hukou Waterfall of the Yellow River	Shapotou Scenic Area	Daming Palace National Heritage Park	Sand Lake Ecotourism Area
	Chaka Salt Lake	Daming Palace National Heritage Park	Bayanbulak Scenic Area	Sand Lake Ecotourism Area	Nalati Scenic Area
	Ami Dongsuo Scenic Area	Kalajun Grassland Scenic Area	Jiangbulake Scenic Area	Urumqi Tianshan Grand Canyon	Nalati Scenic Area

Participant 8 submitted a photo from December 2023 taken at the Jilin Provincial Museum. The top five recommended attractions were all museums, matching the visitor’s preferred type.

Participant 9 uploaded a September 2024 photo of Ta’er Temple. The recommendations were all cultural tourism sites, with Ta’er Temple appearing first, fully matching the visitor’s intended destination.

Participant 10 submitted a photo from June 2024 taken at Guìdé Qingqing Yellow River Scenic Area. The recommendations focused on natural sightseeing sites, including the Qingtongxia Yellow River Grand Canyon and Hukou Waterfall in Shaanxi, consistent with the visitor’s preferred attraction type.

Participant 11 uploaded a May 2025 photo of Chaka Salt Lake. Four of the recommended attractions shared similar natural features, and Chaka Salt Lake ranked first, aligning with the visitor’s ideal site.

Participant 12 submitted a September 2024 photo from Binggou Forest Scenic Area. All recommended sites were natural attractions, with Amu Dongsuo Scenic Area ranked first. Located near Binggou Forest, it closely matched the visitor’s expectation, indicating good recommendation accuracy.

In the image-based recommendation experiments, six participants provided photos from different times, locations, and attraction types. The system successfully recommended matching destinations across the five northwestern provinces of China in all cases, demonstrating reliable performance.

### Recommendation results using EfficientNet + CA + BERT + MLP

Using the EfficientNet + CA + BERT + MLP model, recommendations were generated based on combined text and image inputs from Participants 1–12 (see Table 7). The first four test cases showed stable recommendations that aligned well with the provided text and images. In the last two cases, the results deviated from those of single-modality recommendations due to discrepancies between scenic site conditions and textual descriptions. The system integrated both textual and visual information to provide more suitable recommendations.

**Table 7:** Recommendation results of the EfficientNet + CA + BERT + MLP model.

Text input	Image input	Tourist attraction recommendations (Top1-Top5)				
Prefer places with blue skies, white clouds, and green grass to relax		Qinghai Lake	Sayram Lake	Chaka Salt Lake	Bayanbulak Scenic Area	Nalati Scenic Area
Visiting red tourism sites to experience patriotic feelings		Qinghai Provincial Museum	Qin Shi Huang Mausoleum Museum	Shaanxi History Museum	Gansu Provincial Museum	Xinjiang Khanma Site
Want to experience the performance or exhibition with local characteristics		Taer Monastery	Bayanbulak Scenic Area	Famen Temple Scenic Area	Qinghai Provincial Museum	Yangjialing Revolutionary Site
Low budget, high cost-performance, not pursuing luxury		Pingshan Lake Grand Canyon	Maiji Mountain Scenic Area	Guane Valley Scenic Area	Yangjialing Revolutionary Site	Jiangbulake Scenic Area
Hope for enthusiastic, attentive, and detailed guided explanations		Bayanbulak Scenic Area	Kashgar Old Town	Qinghai Provincial Museum	Qingtongxia Yellow River Grand Canyon	Lanzhou Polar Ocean World
Planning to travel with my child and parents		Yangjialing Revolutionary Site	Jiangbulake Scenic Area	Ami Dongsuo Scenic Area	Urumqi Tianshan Grand Canyon	Maiji Mountain Scenic Area

For example, combining the text “Hope for enthusiastic, attentive, and detailed guided explanations” with sculpture images led the system to recommend indoor attractions such as museums and aquariums, which offer professional guide services. Similarly, integrating “Planning to travel with my child and parents” with grassland or forest images prioritized outdoor sites with educational value, such as red tourism destinations. These examples demonstrate that the model’s multimodal recommendations are more personalized and closely aligned with visitors’ expectations.

### Application example of the T-ECBM recommendation system

To illustrate the practical utility of the proposed system, we present a demonstration from both visitor and tourism planner perspectives. In June 2025, Ms. Li and her family from Shanghai planned a summer trip to Northwest China and used the T-ECBM recommendation system to assist their decision-making. First, Ms. Li registered an account and logged into the mobile application. She then entered her travel details, including the intended season (July–August), budget per person (10,000–15,000 RMB), and companions (two adults and one child), and also uploaded several photos from previous trips to deserts and ancient cities. The system analyzed these textual and visual inputs to capture her personalized preferences. Based on the extracted features, T-ECBM generated a probability-ranked list of candidate attractions, with Dunhuang Mingsha Mountain and Crescent Lake appearing as the top recommendations. After reviewing the suggestions, together with visitor reviews and real-time crowd information provided by the platform, the family selected Mingsha Mountain and Crescent Lake as their destinations. Following the trip, they uploaded photos and shared reviews, which the system incorporated to refine subsequent recommendation outcomes.

This example demonstrates that the T-ECBM system can effectively align recommendations with individual preferences, reduce information search costs, and enhance both decision-making efficiency and overall travel experience.

## Research conclusions and future work

### Research conclusions

This study proposed a novel deep learning-based multimodal tourism recommendation model (T-ECBM) that fuses textual and visual information. By jointly analyzing user review texts and scenic spot images, the model addresses the limitations of single-modality approaches, which often fail to capture both subjective visitor preferences and objective destination features.

On the textual side, BERT was fine-tuned to accurately extract semantic meaning, subjective intentions, and sentiment tendencies from user reviews. On the visual side, EfficientNet enhanced with Coordinate Attention was employed to identify salient scenic elements. The features derived from both modalities were concatenated and passed to a multilayer perceptron, enabling classification-based recommendations. By modeling the recommendation process as a multi-class prediction task, T-ECBM achieved unified joint recommendations while fully leveraging high-dimensional heterogeneous data.

Experimental evaluations demonstrated that T-ECBM substantially outperformed single-modality baselines in terms of Top-1 accuracy, Top-5 accuracy, and F1 score, thereby confirming the advantages of cross-modal fusion for improving recommendation precision and robustness. Even when user intentions were not explicitly stated, the fusion of text and image representations allowed the model to uncover latent preferences, resulting in a purely content-driven recommendation framework. These findings not only validate the effectiveness of deep learning in fusing multimodal data but also provide a replicable technical paradigm for intelligent tourism systems.

From an application perspective, T-ECBM has practical value for enhancing tourism planning and management in Northwest China. The model can support the design of differentiated tourism products by integrating cultural resources with overlapping natural landscapes, thus creating distinctive experiences. It can also help destinations align services and marketing strategies with the needs of specific visitor groups, such as tailoring red tourism sites toward educational travel or improving museum services with more accurate exhibit interpretation. Moreover, it enables the creation of personalized promotional content that highlights historical relics for cultural enthusiasts or natural landscapes for outdoor travelers, thereby increasing marketing effectiveness. Finally, by analyzing correlated visitor interests across nearby attractions, T-ECBM can facilitate regional collaboration—such as jointly promoting Silk Road–themed itineraries across Gansu, Qinghai, Ningxia, and Xinjiang—thereby strengthening the overall influence of Northwest China’s tourism sector.

### Limitations and future work

Despite the promising results, several limitations remain. First, the current model is computationally demanding. Incorporating BERT with over 100 million parameters significantly enhances semantic representation but also requires high computing resources. Future research should therefore explore lightweight architectures and efficient fine-tuning strategies, such as ALBERT, DeBERTaV3, LoRA, or knowledge distillation, to enable deployment in resource-constrained environments.

Second, the study is geographically limited to Northwest China, focusing on plateau lakes, deserts, grasslands, and mountains. Its applicability to other landscapes—such as southern karst formations, islands, tropical rainforests, and culturally diverse regions—has not yet been validated. Expanding datasets to cover national and international destinations, incorporating multilingual reviews and diverse cultural contexts, will be crucial for testing cross-regional adaptability and robustness.

Third, the transferability and interpretability of T-ECBM in domains beyond tourism remain unexplored. Although the model is tailored for travel recommendations, its multimodal fusion strategy has potential relevance for other decision-making scenarios such as healthcare, smart agriculture, transportation, and financial risk management.

Looking ahead, several directions are especially promising. Lightweight deployment techniques such as model pruning, dynamic architectures, and edge computing can reduce inference latency and resource consumption, enabling real-time mobile applications. Dataset expansion at both national and international scales, combined with multilingual and cross-cultural pretraining, will enhance global applicability. Integrating knowledge graphs with causal inference can improve interpretability by clarifying how text sentiment and visual cues contribute to recommendations, while also addressing fairness and robustness. Furthermore, advances in generative multimodal models may support intelligent itinerary planning, automated content creation, and visual explanation, thereby enhancing user experience. Finally, adapting and validating T-ECBM in cross-domain applications will test its generalizability and broaden its industrial value.

By combining model optimization, large-scale data expansion, knowledge integration, and cross-domain deployment, future research can further enhance both the theoretical depth and practical impact of multimodal intelligent recommendation technologies.

## Supplementary Information

Below is the link to the electronic supplementary material.

Supplementary Material 1

## Data Availability

The multimodal dataset used in this study, including tourist textual reviews and images, is publicly available on GitHub at https://github.com/Chenjianfu21/-/tree/master.
