# Supplementary material for: T-ECBM: a deep learning-based text-image multimodal model for tourist attraction recommendation
Source: Sci Rep. 2025 Nov 24;15:41638. doi: 10.1038/s41598-025-25630-z (PMC12644614; doi:10.1038/s41598-025-25630-z)
Supplement: Supplementary file 1 — Supplementary Material 1 [file 41598_2025_25630_MOESM1_ESM.docx]

**Appendix**

**This is a comparison table of Chinese and English scenic spot names**

| **No.** | **Chinese Scenic Spot Name** | **English Scenic Spot Name** |
| --- | --- | --- |
| 0 | 世界魔鬼城 | World Ghost City |
| 1 | 乌鲁木齐天山大峡谷 | Urumqi Tianshan Grand Canyon |
| 2 | 兰州极地海洋世界 | Lanzhou Polar Ocean World |
| 3 | 华山 | Mount Huashan |
| 4 | 华清宫 | Huaqing Palace |
| 5 | 可可托海景区 | Keketuohai Scenic Area |
| 6 | 喀什古城 | Kashgar Old Town |
| 7 | 喀拉峻景区 | Kalajun Grassland Scenic Area |
| 8 | 喀纳斯景区 | Kanas Lake Scenic Area |
| 9 | 嘉峪关关城 | Jiayuguan Fortress |
| 10 | 塔尔寺 | Taer Monastery |
| 11 | 大唐芙蓉园 | Tang Paradise |
| 12 | 大明宫国家遗址公园 | Daming Palace National Heritage Park |
| 13 | 天山天池 | Tianchi Lake of Tianshan |
| 14 | 太白山国家森林公园 | Taibai Mountain National Forest Park |
| 15 | 宁夏水洞沟旅游区 | Shuidonggou Tourist Area |
| 16 | 官鹅沟 | Guane Valley Scenic Area |
| 17 | 崆峒山 | Kongtong Mountain |
| 18 | 巴音布鲁克景区 | Bayanbulak Scenic Area |
| 19 | 平山湖大峡谷 | Pingshan Lake Grand Canyon |
| 20 | 延安宝塔山 | Baota Mountain of Yan’an |
| 21 | 延安革命纪念馆 | Yan’an Revolutionary Memorial Hall |
| 22 | 张掖七彩丹霞旅游景区 | Colorful Danxia scenic spot |
| 23 | 新疆可汗玛基地 | Xinjiang Khanma Site |
| 24 | 曲江海洋极地公园 | Qujiang Ocean Park |
| 25 | 杨家岭革命旧址 | Yangjialing Revolutionary Site |
| 26 | 枣园革命旧址 | Zaoyuan Revolutionary Site |
| 27 | 江布拉克 | Jiangbulake Scenic Area |
| 28 | 沙坡头 | Shapotou Scenic Area |
| 29 | 沙湖生态旅游区 | Sand Lake Ecotourism Area |
| 30 | 法门文化景区 | Famen Temple Scenic Area |
| 31 | 甘肃省博物馆 | Gansu Provincial Museum |
| 32 | 秦始皇帝陵博物院 | Qin Shi Huang Mausoleum Museum |
| 33 | 秦岭野生动物园 | Qinling Wildlife Park |
| 34 | 茶卡盐湖 | Chaka Salt Lake |
| 35 | 葡萄沟 | Grape Valley |
| 36 | 西宁野生动物园 | Xining Wildlife Park |
| 37 | 西安城墙 | The Xi’an Circumvallation |
| 38 | 西安碑林博物馆 | Forest of Stone Steles Museum |
| 39 | 赛里木湖 | Sayram Lake |
| 40 | 那拉提旅游风景区 | Nalati Scenic Area |
| 41 | 镇北堡西部影城 | Zhenbeipu Western Film Studio |
| 42 | 阿咪东索景区 | Ami Dongsuo Scenic Area |
| 43 | 陕西历史博物馆 | Shaanxi History Museum |
| 44 | 陕西黄河壶口瀑布 | Hukou Waterfall of the Yellow River |
| 45 | 青海湖 | Qinghai Lake |
| 46 | 青海省博物馆 | Qinghai Provincial Museum |
| 47 | 青海藏文化博物院 | Qinghai Tibetan Culture Museum |
| 48 | 青铜峡黄河大峡谷 | Qingtongxia Yellow River Grand Canyon |
| 49 | 鸣沙山月牙泉 | Mingsha Mountain and Crescent Lake |
| 50 | 麦积山风景名胜区 | Maiji Mountain Scenic Area |
| 51 | 黄帝陵 | Mausoleum of Yellow Emperor |
